# Concomitant elevations of MMP‐9, NGAL, proMMP‐9/NGAL and neutrophil elastase in serum of smokers with chronic obstructive pulmonary disease

**DOI:** 10.1111/jcmm.13057

**Published:** 2016-12-22

**Authors:** Sarra Bchir, Hela ben Nasr, Sandrine Bouchet, Mohamed Benzarti, Abdelhamid Garrouch, Zouhair Tabka, Santos Susin, Karim Chahed, Brigitte Bauvois

**Affiliations:** ^1^ Unité de recherche UR12ES06Physiologie de l'Exercice et Physiopathologie de l'Intégré au Moléculaire, Biologie, Médecine et SantéFaculté de Médecine de SousseUniversité de SousseSousseTunisia; ^2^ Institut Supérieur de Biotechnologie de MonastirUniversité de MonastirMonastirTunisia; ^3^ Centre de Recherche des CordeliersINSERM UMRS1138Sorbonne Universités UPMC Paris 06Université Paris Descartes Sorbonne Paris CitéParisFrance; ^4^ Assistance Publique des Hôpitaux de ParisParisFrance; ^5^ Service de Pneumo‐AllergologieCHU Farhat HachedSousseTunisia; ^6^ Faculté des Sciences de SfaxUniversité de SfaxSfaxTunisia

**Keywords:** chronic obstructive pulmonary disease, cigarette smoke, GOLD, matrix metalloproteinase, neutrophil elastase, neutrophil gelatinase‐associated lipocalin, serum, systemic inflammation

## Abstract

A growing body of evidence points towards smoking‐related phenotypic differences in chronic obstructive pulmonary disease (COPD). As COPD is associated with systemic inflammation, we determined whether smoking status is related to serum levels of matrix metalloproteinase‐9 (pro‐ and active MMP‐9), neutrophil gelatinase‐associated lipocalin (NGAL) and the proMMP‐9/NGAL complex in patients with COPD. Serum samples were collected in 100 stable‐phase COPD patients (82 smokers, 18 never‐smokers) and 28 healthy adults (21 smokers, 7 never‐smokers). Serum levels of studied factors were measured in ELISA. Our data provide the first evidence of simultaneously elevated serum levels of MMP‐9, NGAL and proMMP‐9/NGAL in COPD smokers. While the triad discriminated between smokers and non‐smokers in the COPD group, MMP‐9 and proMMP‐9/NGAL (but not NGAL) discriminated between smokers with and without COPD. Adjustment for age and smoking pack‐years did not alter the findings. Serum MMP‐9, NGAL and proMMP‐9/NGAL levels were not correlated with the GOLD stage or FEV1 decline. Furthermore, serum levels of neutrophil elastase (NE) and MMP‐3 (but not of IL‐6 and MMP‐12) were also higher in COPD smokers than in healthy smokers before and after adjustment for age and pack‐years. Among COPD smokers, levels of MMP‐9, NGAL and proMMP‐9/NGAL were positively correlated with NE (*P* < 0.0001) but not with the remaining factors. Gelatin zymography detected proMMP‐9 in serum samples of healthy and COPD smoking groups. Our results suggest that associated serum levels of proMMP‐9, NGAL, proMMP‐9/NGAL and NE may reflect the state of systemic inflammation in COPD related to cigarette smoking.

## Introduction

COPD is characterized by chronic inflammation of the airways and the lung parenchyma, leading to progressive, partially reversible or irreversible airway constriction 1, 2. The World Health Organization considers COPD to be the world's fourth leading cause of death 3. The main aetiology of COPD is chronic exposure to tobacco smoke 4, 5. Genetic factors are also important in the development and progression of COPD 6. Impaired lung function is associated with upregulation of chronic inflammatory responses, oxidative stress, airway remodelling and degradation of the extracellular matrix 1, 5. Inflammation and oxidative stress in COPD can be evaluated in blood 7.

Several proteinases are thought to be involved in the pathophysiology of COPD by destroying the extracellular matrix and by regulating inflammation 8, 9, 10. These enzymes include the serine protease NE and MMPs 10, 11, 12. NE is implicated in the initiation and progression of COPD *via* the degradation of elastin and the modulation of inflammatory processes 11, 13. Elevated levels of NE are found in the sputum and blood of patients with COPD 14, 15, 16. When considering the MMPs’ possible involvement in COPD, most attention has been focused on MMP‐9 17. Like most secreted MMPs, MMP‐9 is produced as an inactive zymogen (proMMP‐9, 92 kD); cleavage of the propeptide domain then yields the active MMP‐9 (82 kD) 18. Elevated sputum levels of (pro and active) MMP‐9 are observed in patients with COPD 19, 20 and are correlated with the degree of airflow obstruction 12, 17, 19. Similarly, a number of studies have shown that elevated serum or plasma levels of MMP‐9 are associated with impaired lung function in COPD 12, 17, 21. High serum levels of MMP‐9 are also correlated with the progression of COPD, as assessed by the Global Initiative for Chronic Obstructive Lung Disease (GOLD) stage 22. However, contrasting findings have been reported in other studies of patients with COPD, where (*i*) increased levels of serum MMP‐9 were not related to the decline in FEV1 23, 24, 25 or (*ii*) lower levels of serum MMP‐9 correlated with the disease severity 26. These studies did not take into account the amount of MMP‐9 protein in the latent or active form 12, 17, 21, 22, 23, 24, 25, 26. COPD is heterogeneous, and FEV1 alone does not adequately describe the complexity of this disease 27. A number of ongoing studies are investigating phenotypic differences in COPD 2, 27, 28. A recent study of patients with COPD provided early insight into potential phenotypical differences between lifelong never‐smokers and ever‐smokers 28. The effects of cigarette smoking on MMP‐9 expression and activation are well known 12, 29. In this context, the discrepancies observed for the relationship between MMP‐9 levels and COPD severity may be explained by the use of inappropriately matched healthy and COPD groups that comprised both smokers and never‐smokers 12, 28. Although MMP‐9 blood levels did not differ when comparing current smokers and ex‐smokers with COPD 15, differences between smokers and non‐smokers have been observed in studies of healthy adults 30 and patients with COPD 22, 31.

NGAL is a secreted 25‐kD protein that can bind (*via* a covalent bond) to proMMP‐9 to form a 130‐kD disulphide‐linked heterodimer 18. Like proMMP‐9, NGAL is mainly expressed by activated epithelial and inflammatory cells (monocytes and neutrophils). The NGAL/proMMP‐9 complex is formed within the cell (through an as‐yet unidentified mechanism) prior to secretion 18. NGAL and proMMP‐9/NGAL can be detected in the systemic circulation of healthy adults 18. Furthermore, NGAL has already emerged as an useful biomarker in a wide array of inflammatory diseases 32. Three studies have reported that NGAL levels in plasma 33, serum 34 and induced sputum 35 are higher in patients with COPD than in healthy adults. Whereas Cockayne *et al*. 34 showed that serum NGAL levels are positively correlated with the GOLD stage in patients with COPD (including smokers and non‐smokers), Eagan *et al*. 33 have reported that mean plasma NGAL levels are higher in patients with COPD but fall with increasing GOLD stage.

Separate, ongoing studies are evaluating the inhibition of NE and MMP‐9 (including active MMP‐9 and proMMP‐9) as a novel means of increasing treatment effectiveness in COPD 11, 12, 17, 36, 37. We therefore reasoned that better knowledge of possible correlations between these proteases might be of value in the management of patients with COPD. Hence, we investigated the expression levels of NE, MMP‐9, NGAL and their complex in sera from patients with COPD and in healthy adults, as a function of smoking status. We also sought to determine whether serum levels of these proteins were related to air flow obstruction and the GOLD stage in COPD.

## Materials and methods

### Participants

We defined COPD according to the GOLD criteria. COPD patients with mild, moderate, severe and very severe disease (*n* = 100) were enrolled at the Departments of Pulmonology and Physiology at Farhat Hached Hospital (Sousse, Tunisia). Healthy adults (*n* = 28, the control group) were recruited from the National Blood Transfusion Center (Tunis, Tunisia). All participants lived on the Tunisian central coast. Airflow limitation in COPD is defined as a post‐bronchodilator forced expiratory volume in 1 s (FEV1) to forced vital capacity (FVC) ratio below 70%, and FEV1 reversibility below 12% of the pre‐bronchodilator value after the inhalation of 400 μg of salbutamol. The COPD was staged in accordance with the GOLD guidelines; GOLD I (mild): FEV1 ≥80% predicted; GOLD II (moderate): FEV1 <80% and ≥50% predicted; GOLD III (severe): FEV1 <50% and ≥30% predicted; and GOLD IV (very severe): FEV1 <30% predicted. The COPD was considered to be stable if the patient had not experienced an exacerbation in the 8 weeks prior to the study. The included patients were only taking short‐ or long‐acting β2‐agonists, and they were requested to refrain from taking any medication in the 24 hrs before study measurements were taken. The main exclusion criteria (for both patients and healthy controls) were as follows: neoplastic, metabolic or inflammatory disease, heart failure, current ischaemic symptoms, inhaled/oral corticosteroid treatment or lung disease (other than COPD for patients). The study was approved by the local independent ethics committee (Farhat Hached Hospital, Sousse, Tunisia). All included persons provided their written, informed consent to participation.

### Spirometry

Lung function was evaluated using whole‐body plethysmography (ZAN^®^ 500 Body II, ZAN Messgerate GmbH, Oberthulba, Germany). All measurements complied with the American Thoracic Society's standards. Forced vital capacity and FEV1 were measured from a series of at least three forced expiratory curves. Spirograms were validated for analysis if they were free from artefacts (such as a cough or glottis closure) and if they had good initial sections with an extrapolated volume of less than 5% of the FVC. The exhaled breath manoeuvre lasted for at least 6 sec. After three acceptable manoeuvres, the two highest values for FVC and FEV1 had to be within 0.15 l of each other.

### Blood sample collection and processing

Blood samples were collected within the same period from the patients with COPD and the healthy controls. Serum was immediately separated, aliquoted and frozen at −80°C pending further analyses. Peripheral blood mononuclear cells (PBMCs, *i.e*. monocytes, T lymphocytes and B lymphocytes) were isolated from the samples using Histopaque^®^‐1077 density gradient centrifugation. Cell pellets were frozen at −80°C until RNA extraction and analysis.

### Qualitative gelatin zymography

Serum expression profiles of MMP‐2/MMP‐9 (pro‐ and active forms) and proMMP‐9/NGAL were analysed using 7.5% (w/v) SDS–polyacrylamide gels containing 0.1% gelatin (w/v), as described in 38. The MMPs’ gelatinolytic activities were visualized as transparent bands on the background of Eza‐blue‐stained gelatin (Sigma‐Aldrich, Saint Louis, Mo, USA). IMAGEJ software (version 1.47) (National Institute of Health, USA) was used to analyse the bands after acquisition with an Appligen densitometer (Oncor, Illkirch, France). As previously described, culture supernatant (treated with aminophenylmercuric acid) from phorbol myristate acetate‐activated U937 monocyte‐derived cells was used as a positive control for pro‐ and active MMP‐2/MMP‐9 proteins, and primary leukaemic B cells were used as a positive control for proMMP‐9 and the proMMP‐9/NGAL complex 18. The assay's sensitivity for MMP‐2/MMP‐9 gelatinolytic activity is 25 ng/ml.

### RNA isolation, cDNA synthesis and PCR

RNA was extracted from PBMCs, and cDNA was synthesized as described in 38. The cDNAs for human β2‐microglobulin (165 bp), MMP‐2 (225 bp), MMP‐9 (296 bp), MMP‐12 (1069 bp), NGAL (479 bp) and IL‐6 (562 bp) were amplified in PCRs using the published primer sequences (Eurogentec, Angers, France), as previously described in detail 38, 39, 40, 41, 42, 43, 44. The annealing temperature for primers was 57°C. The PCR products were visualized by electrophoresis in a 1.8% agarose gel containing 0.2 μg/ml ethidium bromide. The band intensity of the PCR products of interest (measured in an Appligen densitometer (Oncor) and analysed using IMAGEJ 1.47 software) was normalized against that of β2‐microglobulin (internal control).

### Genotyping of MMP‐9 (the C‐1562T polymorphism)

Genomic DNA was extracted from PBMCs in a salting‐out procedure, as previously described 45. The MMP‐9 genotyping was determined by PCR‐restriction fragment length polymorphism. The primers used for amplifying the MMP‐9 C‐1562T polymorphism were 5′‐GCCTGGCACATAGTAGGCCC‐3′ (forward) and 5′‐CTTCCTAGCCAGCCGGCATC‐3′ (reverse). The PCRs were performed as previously described 45. The PCR products of C‐1562T polymorphism sites were digested with restriction enzymes and separated by electrophoresis on a 2% agarose gel. The presence of an SphI site was indicated by the cleavage of the 435‐bp amplified product into two fragments (247 and 188 bp, respectively) 45.

### ELISAs

Levels of total (pro and active) MMP‐2, MMP‐3, MMP‐7 and MMP‐9, NGAL, proMMP‐9/NGAL (130 kD), IL‐6 and TNF‐α were determined using commercial ELISA kits (R&D Systems, Abingdon, UK). An ELISA for NE was purchased from Abcam (Paris, France), and an ELISA for (pro and active) MMP‐12 was obtained from Boster Biological Technology (Pleasanton, CA, USA). The minimum detectable dose was 33 pg/ml for MMP‐2 and MMP‐12, 100 pg/ml for MMP‐9, 9–16 pg/ml for MMP‐3, MMP‐7, NGAL and proMMP‐9/NGAL, 1–2 pg/ml for IL‐6 and NE and 0.1 pg/ml for TNF‐α. The coefficients of variation of the intra‐ and interassay values were, respectively, 1.9–2.9% and 6.9–7.9% for MMP‐9, 3.6–7% and 6.5–7% for MMP‐2, 5.7–6.4% and 7–8.6% for MMP‐3, 3.4–5.1% and 4.1–4.6% for MMP‐7, 3.6–4.4% and 5.6–7.9% for NGAL, 2.3–4.1% and 5.1–7.6% for proMMP‐9/NGAL, 1.6–4.2% and 3.3–6.4% for IL‐6, 3.1–8.7% and 7.2–10.4% for TNF‐α, 4.8% and 5.6% for NE and 4.5–6% and 5.4–7.2% for MMP‐12. All assays were performed in accordance with the manufacturer's instructions. Wide variations in serum NGAL values exist within the healthy population (median values ranging from 0.6 to 199 ng/ml) 46, 47, 48, 49, 50, 51, 52, 53, 54. Herein, the median NGAL level in the whole healthy population (including smokers and non‐smokers) was 11.09 ng/ml (with an interquartile range of 6.23–16.74).

### Statistical analysis

Data were expressed as the mean ± S.E.M. or the median (interquartile range, IQR). Groups were compared using Student's *t*‐test, the Mann–Whitney *U*‐test or an analysis of variance. Spearman's correlation coefficient was calculated as a guide to the relationships between MMP, NGAL, proMMP‐9/NGAL, NE and IL‐6 levels and respiratory parameters. An analysis of covariance was used to assess MMPs, proMMP‐9/NGAL, NGAL, NE and IL‐6 levels in healthy smokers (hereafter referred to as the healthy‐S group) and COPD smokers (the COPD‐S group) as a function of age and pack‐years. All statistical analyses were performed using SPSS^®^ software (version 17.0 for Microsoft Windows, SPSS Inc., Chicago, IL, USA) and GraphPad Prism software (version 6.04; GRAPHPAD Software, La Jolla, CA, USA). The threshold for statistical significance was set to *P* < 0.05.

## Results

### Characteristics of the study population

A total of 128 participants (28 controls and 100 patients with COPD) were included in the study. The participants’ demographic and clinical characteristics are summarized in Table 1. The mean ± S.E.M. number of pack‐years was significantly higher in the COPD‐S group than in the healthy‐S group (46.66 ± 3.2 *versus* 17.39 ± 12.89, respectively; Table 1). We analysed two COPD smoker subgroups: 43 GOLD stage I/II patients (9 GOLD I + 34 GOLD II) and 39 GOLD stage III/IV patients (34 GOLD III + 5 GOLD IV). All the healthy controls (regardless of their smoking status) had a normal FEV1% predicted >90. All the patients with COPD (regardless of their smoking status) had mild‐to‐severe airflow obstruction, and all spirometric values were significantly lower in the COPD group (and the GOLD smoker subgroups I/II and III/IV) than in the healthy individuals (Table 1).

**Table 1:** Demographic and clinical characteristics of healthy individuals and patients with COPD

	Healthy		COPD smokers		GOLD stage	
	Non‐smokers	Smokers	Non‐smokers	Smokers	GOLD I/II	GOLD III/IV
	*n* = 7	*n* = 21	*n* = 18	*n* = 82	*n* = 43	*n* = 39
Age(years)	52.09 ± 2.1	57.81 ± 1.9	54.83 ± 4	63.59 ± 1.35	63.23 ± 1.91	64 ± 1.94
Male (%)	57.1	100	33.3	98.8	100	97.4
BMI	26.78 ± 1.22	27.02 ± 0.91	26.88 ± 2.02	24.44 ± 0.54	24.18 ± 0.6	24.72 ± 0.93
Smokers/ex‐smokers	0	17/4	0	42/40	25/18	17/22
Pack‐years	0	17.39 ± 12.89	0	46.66 ± 3.2	49.25 ± 4.88	43.8 ± 4.07
FEV1	3.08 ± 0.39	3.02 ± 0.17	1.45 ± 0.17	1.55 ± 0.07	2 ± 0.1	1.06 ± 0.04
FEV1% pred	92.83 ± 1.07	93.56 ± 2.46	60.43 ± 5.25	54.42 ± 2.26	69.49 ± 2.35	37.73 ± 1.25
FVC	3.84 ± 0.46	3.64 ± 0.2	2.29 ± 0.26	3.2 ± 0.37	3.98 ± 0.67	2.34 ± 0.09
FVC % pred	98.17 ± 2.45	94.19 ± 2.58	79.57 ± 6.17	79.12 ± 2.28	91.34 ± 2.72	65.59 ± 2.19

Data are presented as mean ± S.E.M. COPD: chronic obstructive pulmonary disease; BMI: body mass index [weight (kg) / height^2^ (m^2^)].

Pack‐years (number of cigarettes smoked per day × number of years smoked) /20; FEV1: forced expiratory volume in 1 s, % pred: per cent of predicted value; FVC: forced vital capacity.

### Serum levels of MMP‐9, proMMP‐9/NGAL and NGAL are elevated in smokers with COPD

**Figure 1:**
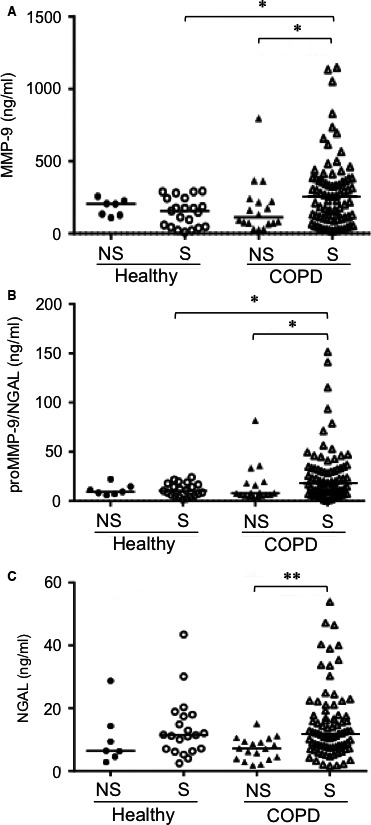
Serum concentrations of (pro and active) MMP‐9, NGAL and the proMMP‐9/NGAL complex in healthy individuals and patients with COPD. ELISAs were performed on seven healthy non‐smokers, 21 healthy smokers, 18 COPD non‐smokers and 82 COPD smokers. (**A**) Pro‐ and active MMP‐9, (**B**) proMMP‐9/NGAL and (**C**) NGAL protein levels were determined. *P*‐values were calculated using a Mann–Whitney *U*‐test. *P < 0.05, **P < 0.01. NS: non‐smoker; S: smoker; COPD: chronic obstructive pulmonary disease.

The data on healthy controls and patients with COPD were categorized according to smoking status. All the serum assays of (pro and active) MMP‐9, NGAL and proMMP‐9/NGAL were performed at the same time‐point (Fig. 1). Serum MMP‐9 levels were significantly higher in the COPD‐S group (258.68 ng/ml) than in the healthy‐S group (156.95 ng/ml; *P* = 0.04 *versus* COPD‐S) and the COPD‐NS group (111.38 ng/ml; *P* = 0.05 *versus* COPD‐S; Fig. 1A). Similarly, the level of proMMP‐9/NGAL was markedly higher in the COPD‐S group (17.9 ng/ml) than in the healthy‐S group (10.42 ng/ml; *P* = 0.01 *versus* COPD‐S) and the COPD‐NS group (7.87 ng/ml; *P* = 0.02 *versus* COPD‐S; Fig. 1B). When adjusted for age and pack‐years, both MMP‐9 and proMMP‐9/NGAL levels remained significantly associated with smoking status in COPD when compared with the healthy‐S group (MMP‐9: *P* adjusted for age = 0.001, and *P* adjusted for age and pack‐years = 0.003; proMMP‐9/NGAL: *P* adjusted for age = 0.014, and *P* adjusted for age and pack‐years = 0.017; Table S1). There were no significant differences in either MMP‐9 or complex levels when comparing the healthy‐NS, healthy‐S and COPD‐NS groups (Fig. 1A and B; *P* > 0.28). Likewise, serum NGAL levels were significantly higher in the COPD‐S group than in the COPD‐NS group (11.9 *versus* 7.1 ng/ml, respectively; *P* = 0.001; Fig. 1C). However, NGAL expression did not differ markedly when comparing the COPD‐S and healthy‐S groups (11.9 *versus* 11.4 ng/ml, respectively; *P* = 0.81; *P* adjusted for age = 0.77; *P* adjusted for age and pack‐years = 0.92; Table S1). Moreover, there were no significant differences in the serum levels of MMP‐9, proMMP‐9/NGAL or NGAL between the two COPD‐S subgroups, .that is GOLD stage I/II and GOLD stage III/IV (Table 2). FEV1% predicted was not correlated with levels of these analytes (*P* ≥ 0.33).

**Table 2:** Serum levels of NE, IL‐6, (pro and active) MMP‐9, proMMP‐9/NGAL and NGAL in COPD smokers according to disease stages

Study parameters	GOLD I/II	GOLD III/IV	*P*‐value
MMP‐9 (ng/ml)	259.93 (99.09–350.28)	243.31 (108.25–392.51)	0.63
ProMMP‐9/NGAL (ng/ml)	18.59 (7.29–34.99)	17.35 (9.93–33.17)	0.66
NGAL (ng/ml)	11.85 (8.08–21.05)	12.1 (7.18–15.97)	0.56
NE (ng/ml)	63.69 (47.15–93.48)	76.93 (61.15–103.28)	0.24
IL‐6 (pg/ml)	3.24 (2.44–6.2)	3.03 (2.29–4.09)	0.64

Data are presented as median (interquartile range). Comparisons were made using the Mann–Whitney *U*‐test.

MMP: matrix metalloproteinase; NE, neutrophil elastase; NGAL, neutrophil gelatinase‐associated lipocalin; IL‐6, interleukin‐6. MMP‐9 ELISA determines pro‐ and active MMP‐9 concentrations in serum.

### Serum levels of NE are elevated in patients with COPD, regardless of smoking status

As serum NE and IL‐6 levels have been found to be associated with COPD 15, 16, 55, 56, they were used as positive controls for COPD in an analysis of the four subgroups. The mean serum levels of NE and IL‐6 were significantly higher in the COPD‐S group than in the healthy‐S group (NE: 69.29 *versus* 46.89 ng/ml, respectively, *P* = 0.006; IL‐6: 3.13 *versus* 2.25 pg/ml, respectively; *P* = 0.001, both in a Mann–Whitney *U*‐test). However, there were no significant differences in NE or IL‐6 levels between the COPD‐S and COPD‐NS subgroups (*P* > 0.06). After adjustment for age and pack‐years, NE levels (but not IL‐6 levels) were still significantly higher in the COPD group than in the healthy controls (NE: *P* adjusted for age = 0.014; *P* adjusted for age and pack‐years = 0.04; Table S1). Serum levels of both NE and IL‐6 did not differ when comparing the two COPD‐S subgroups (GOLD stage I/II *versus* GOLD stage III/IV; Table 3). FEV1% predicted was negatively correlated with serum NE (*r* = −0.223, *P* = 0.03) but was not correlated with IL‐6 (*P* = 0.7).

**Table 3:** Serum levels of (pro and active) MMP‐2, MMP‐3, MMP‐7 and MMP‐12 in healthy and COPD smokers according to disease stages

Parameters	Healthy smokers	COPD smokers	*P* *	GOLD I/II	GOLD III/IV	*P* **
MMP‐2 (ng/ml)	110.75 (97.24–128.24)	105.21 (86.18–119.22) –	**0.02**	98.21(83.13–113.76)	113.14(86.38–130.09)	0.07
MMP‐3 (ng/ml)	1.44 (1.29–1.79)	2.45 (1.49–3.44)	**0.01**	1.78(1.34–2.59)	3.24(2.22–3.82)	**0.01**
MMP‐7 (ng/ml)	0.14 (0.12–0.16)	0.19 (0.09–0.36)	0.64	0.17(0.09–0.27)	0.25(0.09–0.43)	0.3
MMP‐12 (ng/ml)	171.65 (150.08–239.44)	341.55 (202.9–526.85)	**0.03**	387.32(213.02–531.25)	289.79(182.66–440.14)	0.38

Data are presented as median (interquartile range). Bold *P*‐value represents statistical significance. *Comparisons between healthy smokers and COPD smokers and **comparisons between GOLD I/II and GOLD III/IV were made using the Mann–Whitney *U*‐test. MMP, matrix metalloproteinase. ELISAs determine pro‐ and active MMP concentrations in serum.

### Intercorrelations between serum levels of MMP‐9, NGAL, proMMP‐9/NGAL and NE in COPD smokers

**Figure 2:**
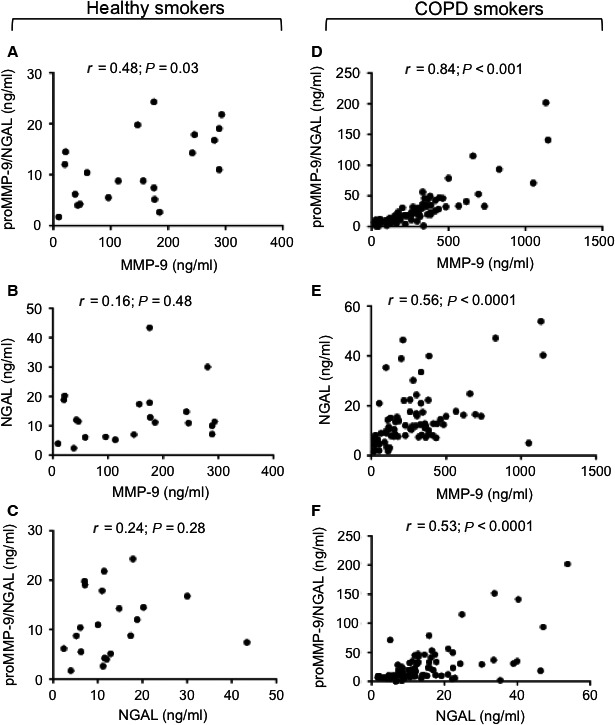
Correlations between serum (pro and active) MMP‐9, NGAL and proMMP‐9/NGAL levels in healthy smokers (*n* = 21) (**A‐C**) and COPD smokers (*n* = 82) (**D‐F**). Spearman's correlation coefficient (*r*) and the *P*‐value are shown. Spots are superposed in the COPD group.

We first investigated correlations between serum levels of (pro and active) MMP‐9, NGAL and the proMMP‐9/NGAL complex in the COPD‐S and healthy‐S groups. In the healthy‐S subgroup, we observed a positive correlation between MMP‐9 and proMMP‐9/NGAL levels (*r* = 0.48, *P* = 0.03; Fig. 2A), whereas no correlations were found for MMP‐9 and NGAL (*r* = 0.16, *P* = 0.48; Fig. 2B) or for NGAL and the proMMP‐9/NGAL complex (*r* = 0.24, *P* = 0.28; Fig. 2C). In contrast, all the studied parameters were significantly correlated in the COPD‐S group (Fig. 2D–F): there were positive correlations between MMP‐9 and the proMMP‐9/NGAL complex (*r* = 0.84, *P* < 0.0001; Fig. 2D), between MMP‐9 and NGAL (*r* = 0.56, *P* < 0.0001; Fig. 2E) and between NGAL and the proMMP‐9/NGAL complex (*r* = 0.53, *P* < 0.0001; Fig. 2F).

**Figure 3:**
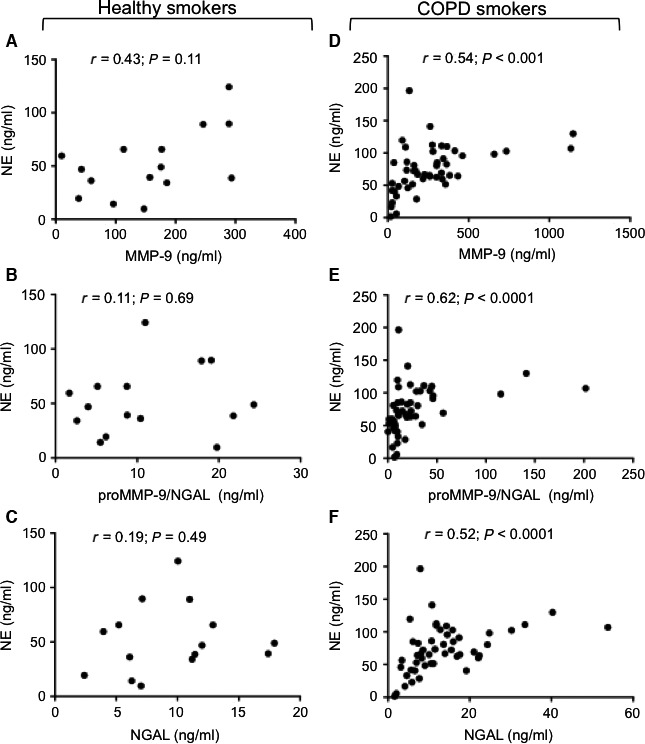
Intercorrelations between serum (pro and active) MMP‐9, NGAL and proMMP‐9/NGAL levels in healthy smokers (*n* = 15) (**A‐C**) and smokers with COPD (*n* = 46) (**D‐F**). Spearman's correlation coefficient (*r*) and the *P*‐value are shown. Spots are superposed in the COPD group.

Next, we tested for associations between serum NE on the one hand and serum MMP‐9, NGAL and the proMMP‐9/NGAL complex on the other in the COPD‐S and healthy‐S groups (Fig. 3A–F). In the healthy‐S group, NE was not correlated with MMP‐9 (*r* = 0.43, *P* = 0.11; Fig. 3A), NGAL (*r* = 0.11, *P* = 0.169; Fig. 3B) and the proMMP‐9/NGAL complex (*r* = 0.19, *P* = 0.49; Fig. 3C). However, in the COPD‐S group, the NE level was strongly correlated with all three analytes, that is MMP‐9 (*r* = 0.54, *P* < 0.0001; Fig. 3D), NGAL (*r* = 0.62, *P* < 0.0001; Fig. 3E) and proMMP‐9/NGAL (*r* = 0.52, *P* < 0.0001; Fig. 3F). In contrast, IL‐6 levels were not correlated with serum levels of MMP‐9, NGAL, proMMP‐9/NGAL or NE in the COPD‐S group (Table S2).

### High MMP‐3 and low MMP‐2 serum levels in COPD smokers

We examined the serum levels of (pro and active) MMP‐2/MMP‐3/MMP‐7 and MMP‐12 proteins in the healthy‐S and COPD‐S groups. The serum level of MMP‐2 was lower in the COPD‐S group than in the healthy‐S group (105.21 *versus* 110.75 ng/ml, respectively; *P* = 0.02; Table 3). In contrast, serum levels of MMP‐3 and MMP‐12 (but not MMP‐7) were significantly higher in COPD smokers than in healthy smokers (MMP‐3: 2.45 *versus* 1.44 ng/ml, respectively, *P* = 0.01; MMP‐12: 341.55 *versus* 171.65 ng/ml, respectively, *P* = 0.03; Table 3). After adjustment for age and pack‐years, MMP‐2 and MMP‐3 levels were still significantly higher in the COPD‐S group than in the healthy‐S group (Table S1). There were no significant differences in MMP levels between the healthy‐NS, healthy‐S and COPD‐NS subgroups (data not shown). Moreover, the level of serum MMP‐3 (but not the other MMPs) was higher in smoking patients with advanced disease (GOLD stages III/IV) than in those with mild–moderate disease (GOLD stages I/II; Table 3). FEV1% predicted was negatively correlated with MMP‐3 (*r* = −0.37, *P* = 0.01) but was not correlated with levels of the other MMPs (*P* ≥ 0.09). Furthermore, the correlations between the serum levels of MMP‐2, MMP‐3, MMP‐12 on one the hand and MMP‐9, NGAL, the complex and NE on the other were also evaluated in the COPD‐S group: we identified a single, negative correlation between MMP‐2 and NE (*r* = −0.293, *P* = 0.04; Table S2).

### The proforms of MMP‐2/MMP‐9 are present in sera of healthy smokers and COPD smokers

**Figure 4:**
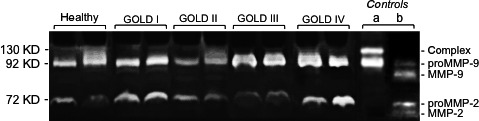
Levels of proMMP‐2 and proMMP‐9 in serum samples from healthy and COPD smokers**.** Representative gelatin zymograms of two samples from each group are shown. Controls include (a) proMMP‐9 and proMMP‐9/NGAL, and (b) proMMP‐2/MMP‐9 and active MMP‐2/MMP‐9. Expression profiles of proMMP‐2/MMP‐9 and the proMMP‐9/NGAL complex in sera (5 μl) for healthy smokers and COPD smokers (GOLD I‐IV).

The gelatinases MMP‐2 and MMP‐9 are known to be expressed as proforms (72 and 92 kD, respectively) in the serum of healthy individuals 57. Using qualitative gelatin zymography, we demonstrated that the proforms of MMP‐2 and MMP‐9 were most strongly present in sera from the healthy‐S and COPD‐S subgroups (20 and 42 samples tested, respectively). Representative examples of zymography products are shown in Figure 4. In contrast, the proMMP‐9/NGAL complex (130 kD) was barely or not detectable, although this could be explained by the zymographic assay's limit of detection (Fig. 4).

### Elevated levels of MMP‐9 transcripts in PBMCs from COPD smokers are correlated with the GOLD stage and the degree of airflow obstruction

**Figure 5:**
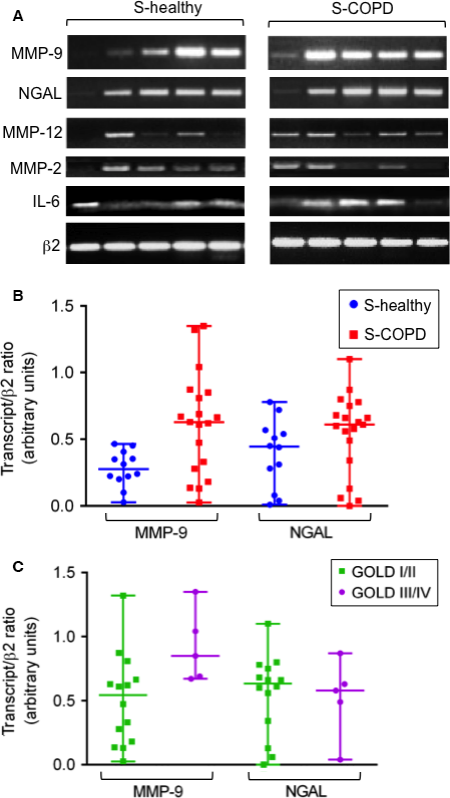
RT‐PCR analysis of MMP‐2/ MMP‐9/MMP‐12, NGAL and IL‐6 transcripts in PBMCs from healthy and COPD smokers. (**A**) Representative RT‐PCR results for five samples from healthy and COPD smokers. The sample's cDNA content was normalized against the value for β2‐microglobulin. (**B** & **C**) Data are expressed as the ratio between the analyte transcript and the β2‐microglobulin transcript. Values are reported as the median (IQR). (**B**) Levels of MMP‐9 and NGAL transcripts in samples from healthy‐S (*n* = 12) and COPD‐S (*n* = 19) samples. (**C**) Levels of MMP‐9 and NGAL transcripts by GOLD stage groups (GOLD I/II, *n* = 14; GOLD III/IV, *n* = 5).

We next used a semiquantitative RT‐PCR method to profile the expression of MMP‐2/MMP‐9/MMP‐12, NGAL and IL‐6 transcripts in PBMCs isolated from blood samples of 19 COPD‐S patients and 12 healthy‐S participants (for the patients’ characteristics, see the Table S3). As shown in Figure 5A**,** PBMCs from both groups expressed detectable levels of MMP‐9, NGAL, MMP‐12, MMP‐2 and IL‐6 transcripts (Fig. 5A). The median level of MMP‐9 transcripts appeared to be higher in the COPD‐S group than in the healthy‐S group (Fig. 5B); in contrast, the two groups did not differ significantly in terms of NGAL, MMP‐2, MMP‐12 and IL‐6 expression levels (see Fig. 5B for NGAL and Fig. S4 for MMP‐2, MMP‐12 and IL‐6). Furthermore, the level of MMP‐9 transcripts was found to be higher in patients with advanced disease (GOLD stages III/IV) than in those with mild–moderate disease (GOLD stages I/II; Fig. 5C). The differences observed for MMP‐9 will have to be confirmed by qRT‐PCR. Furthermore, FEV1% predicted was negatively correlated with the level of MMP‐9 transcripts (*r* = −0.514, *P* = 0.02). In contrast, FEV1% predicted was not correlated with levels of NGAL, MMP‐2, MMP‐12 or IL‐6 transcripts in the COPD‐S group (*P* ≥ 0.1).

### Lack of an association between the C‐1562T polymorphism and serum MMP‐9 levels in COPD smokers

We previously reported that serum MMP‐9 levels were significantly higher in COPD patients with the CT genotype (regardless of smoking status) than in healthy controls 45. In the present study, we analysed the association between the C‐1562T polymorphism and serum MMP‐9 levels in COPD non‐smokers *versus* smokers. The proportions of patients with the C‐1562T genotype in COPD‐NS and COPD‐S groups were within the same range (6% *versus* 12.6%, respectively), and the respective serum levels of MMP‐9 were similar (365.38 *versus* 352.03 ng/ml, respectively).

## Discussion

Serum MMP‐9 and NGAL merit further investigation as putative, specific biomarkers of smoking status in COPD. To the best of our knowledge, the potential value of the proMMP‐9/NGAL complex in the pathogenesis of COPD has not previously been investigated. In the present study of a well‐characterized cohort of healthy and COPD smokers and non‐smokers, we observed that serum levels of MMP‐9, NGAL and proMMP‐9/NGAL were higher in COPD smokers than in COPD non‐smokers. Whereas levels of MMP‐9 and the proMMP‐9/NGAL complex discriminate between COPD smokers and healthy smokers, NGAL appears to be a marker of smoking status (irrespective of disease status). However, these relationships would need to be confirmed in a more extensive study of a larger cohort of healthy non‐smokers and smokers. In healthy adults, serum NGAL is correlated with age 58, 59, but serum MMP‐9 may not be 60, 61, 62. In the present study, age‐corrected serum levels of MMP‐9 and proMMP‐9/NGAL were correlated with smoking status in patients with COPD but not in healthy smokers. Moreover, there was a lack of positive correlation between smoking history (pack‐years) and these two markers. Hence, both MMP‐9 and proMMP‐9/NGAL may be potential serum biomarkers of smoking status in COPD. There were no significant differences in serum levels of MMP‐9, NGAL and proMMP‐9/NGAL when comparing GOLD stages. Moreover, the levels of these analytes were not related to the severity of COPD (as measured by the degree of airflow obstruction). Our findings agree with those of three studies in which serum MMP‐9 levels in COPD smokers were not related to a decline in FEV1% predicted 23, 24, 25. Interestingly, our COPD group as a whole (irrespective of smoking status, *n* = 100) displayed higher serum MMP‐9 levels than the 28 healthy adults (215.45 *versus* 166 ng/ml, respectively; *P* = 0.04). In patients with COPD, MMP‐9 levels were significantly correlated (*r* = −0.223, *P* = 0.03) with the degree of airway obstruction. This observation emphasizes the bias caused by underestimating the impact of cigarette smoking on MMP‐9 levels 12.

Our results highlighted the previously reported COPD‐related elevations in serum levels of NE and MMP‐3, irrespective of the patients’ smoking status 15, 16, 22, 25, 56. There are two conflicting literature reports on the associations between NE levels and FEV1 in COPD 15, 16. In the present study, NE and MMP‐3 were negatively correlated with FEV1, but only MMP‐3 levels increased with the disease severity (GOLD stage). Although it is well known that serum IL‐6 levels are elevated in COPD 15, 16, 55, 56, our present study revealed that this elevation is age‐ and pack‐years‐related. As previously reported for the COPD population as a whole 15, 25, 56, 63, our study demonstrated the absence of a correlation between serum IL‐6 and poor lung function in COPD smokers. Given the emerging role of MMP‐12 in the pathogenesis of COPD 36, 64, we probed the putative relationship between COPD and serum MMP‐12. Elevated levels of MMP‐12 have been observed in bronchoalveolar macrophages and sputum samples from patients with COPD 36, 65. Our data indicate that serum MMP‐12 levels are elevated in patients with COPD (irrespective of smoking status) but are also age dependent. Moreover, serum MMP‐12 levels did not correlate with airflow obstruction and disease severity (GOLD stage).

The present study is the first to highlight the interrelationships between serum levels of MMP‐9, NGAL, the proMMP‐9/NGAL complex and NE in COPD smokers. There is growing evidence to suggest that the release of specific factors by blood monocytes and neutrophils contributes to chronic inflammation in COPD 12, 13, 17. Whereas both monocytes and neutrophils synthesise significant amounts of MMP‐9, NGAL and proMMP‐9/NGAL complex (the formation of which depends on the intracellular concentrations of NGAL and proMMP‐9), NE is most abundant in neutrophils. Blood cell activation and release is influenced by cigarette smoking and pro‐inflammatory cytokines [such as tumour necrosis factor alpha (TNF‐α)]. The TNF‐α system (including TNF‐α, TNF receptors and the NF‐κB signalling pathway) is activated in patients with COPD 66. Cigarette smoke is linked to NF‐κB activation and NF‐κB‐mediated pro‐inflammatory cytokine release 5, 67. NF‐κB is known to induce the transcription of MMP‐9 and NGAL 32, 68. TNF‐α induces the release of NE by human neutrophils 69. Other studies have observed the synthesis and release of MMP‐9 and NE by inflammatory cells exposed to cigarette smoke condensate 70, 71. Thus, the concomitantly elevated levels of MMP‐9, NE and NGAL might be due (at least in part) to TNF‐α's ability to activate the expression and/or release of these proteins by blood cells. However, the ELISA results showed that median serum levels of TNF‐α were similar in COPD smokers (1.24 pg/ml, 0.73–1.82), COPD non‐smokers (1.22 pg/ml, 0.74–1.24) and healthy smokers (0.89 pg/ml, 072–3.31). This rules out the involvement of serum TNF‐α in elevated serum levels of MMP‐9, NGAL and NE in COPD smokers. The contribution of other inflammatory factors to the upregulation of these proteins remains to be determined. Alternatively, genetic variations (such as promoter polymorphisms) may influence the expression levels of these proteins. To the best of our knowledge, promoter polymorphisms of NE and NGAL are not more prevalent or less prevalent in COPD than in the general population. In contrast, the C‐1562T polymorphism in the MMP‐9 promoter is associated with COPD in various populations 45, 72, 73, 74. Our previously published data suggested that the C‐1562T polymorphism could affect MMP‐9 protein expression in a cohort of patients with COPD, although we did not consider smoking status 45. Here, we analysed the association between the C‐1562T polymorphism and serum MMP‐9 levels in both COPD smokers and non‐smokers. We did not observe any significant associations between serum MMP‐9 levels, the C‐1562T polymorphism and smoking status. It has been shown that a second promoter polymorphism in the MMP‐9 gene (90(CA)14–24) affects blood MMP‐9 levels in healthy adults and obese patients 68, 75. Furthermore, five polymorphisms in the NE promoter region have been linked to the risk of lung cancer 76. However, no relationships between the latter polymorphisms and COPD have yet been reported.

Gene expression profiles in blood monocytes may serve as diagnostic markers in COPD 77. Our RT‐PCR experiments detected the expression of MMP‐2, MMP‐9, MMP‐12, NGAL and IL‐6 transcripts by the monocyte subpopulation in PBMCs. We observed significantly higher MMP‐9 transcript levels in PBMCs from COPD smokers than in cells from healthy smokers. These differences must now be confirmed in a qRT‐PCR study. The elevated serum MMP‐9 level in COPD smokers could be explained (at least in part) by upregulation of MMP‐9 transcripts in monocytes. Our observation agrees with an earlier study in which MMP‐9 release by blood monocytes was accentuated in patients with COPD 78. In agreement with the study by Abd El‐Fatah *et al*. 79, levels of MMP‐9 transcript in PBMCs were correlated with airflow obstruction and disease severity (as assessed by the GOLD stage). The relative contribution of blood neutrophils to elevated serum levels of NGAL and NE remains to be determined. Lastly, it was recently reported that serum MMP‐9 levels are correlated with blood cell counts in both never‐smokers and current smokers 31. It now remains to be seen whether the elevated levels of MMP‐9, NGAL, MMP‐9/NGAL and NE in COPD smokers observed in the present study is associated with elevated blood cell counts.

## Conclusion

Our study constitutes a step forward in the identification and validation of serum biomarkers for COPD in cohort trials. Serum MMP‐9, NGAL, proMMP‐9/NGAL and NE appear to be reliable, interlinked biomarkers of smoking status in COPD. It remains to be determined whether the association between these biomarkers reflects a causal relationship. In healthy adults and patients with COPD, serum MMP‐9 is mainly present as proMMP‐9. It is known that NE can activate proMMP‐9 80. Clinical researchers have sought to target the catalytic activities of NE and active MMP‐9 (separately) in COPD 11, 12, 17, 36, 37. Moreover, the recognition of NE, NGAL and MMP‐9 (as its proform) as outside‐in signalling molecules 13, 18, 57 has prompted the development of novel cognate inhibitors (including function‐blocking antibodies and peptides). Simultaneously targeting proMMP‐9 and NE might be a potentially effective strategy (alone or combined with other approaches) for treating COPD smokers.

## Authors’ contribution

S. Bchir collected clinical samples, designed the study, analysed and interpreted data and revised the manuscript for critical content. H. ben Nsar collected clinical samples. S. Bouchet provided critical technical support in experimental design and data analysis. M. Benzarti, A. Garrouch and Z. Tabka collected clinical samples and recorded the demographic and clinical characteristics of participants. S. Susin provided critical enhancement of data and revised the manuscript for critical content. K. Chahed contributed to the study design and revised the manuscript for critical content. B. Bauvois contributed to experimental design, analysed and interpreted data, performed a literature review and wrote up the main structure of the manuscript. All authors read and approved the submitted manuscript.

## Conflicts of interest

The authors declare no conflict of interests.

## Supporting information


**Table S1** *P*‐values of serum concentrations of MMPs, NGAL, proMMP‐9/NGAL, NE and IL‐6 between healthy smokers and COPD smokers, before and after adjustment for age and pack years.

**Table S2** Mutual correlations among (pro and active) MMPs, proMMP‐9/NGAL, NGAL, NE and IL‐6 in serum from COPD smokers.

**Table S3** Demographic and clinical characteristics of healthy and COPD smokers.

**Figure S1** RT‐PCR analysis of MMP‐2/‐12 and IL‐6 transcripts in PBMCs from healthy smokers and COPD smokers.
