# Supplementary material for: Concomitant elevations of MMP‐9, NGAL, proMMP‐9/NGAL and neutrophil elastase in serum of smokers with chronic obstructive pulmonary disease
Source: J Cell Mol Med. 2016 Dec 22;21(7):1280–91. doi: 10.1111/jcmm.13057 (PMC5487915; doi:10.1111/jcmm.13057)
Supplement: Supplementary file 1 — Table S1 P‐values of serum concentrations of MMPs, NGAL, proMMP‐9/NGAL, NE and IL‐6 between healthy smokers and COPD smokers, before and after adjustment for age and pack years. [file JCMM-21-1280-s001.docx]

**Supplemental data 1** P-values of serum concentrations of MMPs, NGAL, proMMP-9/NGAL, NE and IL-6 between healthy smokers and COPD smokers, before and after adjustment for age and pack years

| Parameters | p-value | p-value adjusted for age | p-value adjusted for  age and pack years |
| --- | --- | --- | --- |
| MMP-9 | 0.04 | 0.001 | 0.003 |
| proMMP-9/NGAL | 0.01 | 0.014 | 0.017 |
| NGAL | 0.81 | 0.77 | 0.92 |
| MMP-2 | 0.02 | 0.017 | 0.023 |
| NE | 0.006 | 0.014 | 0.04 |
| IL-6 | 0.001 | 0.31 | 0.25 |
| MMP-3 | 0.01 | 0.036 | 0.035 |
| MMP-7 | 0.64 | 0.81 | 0.91 |
| MMP-12 | 0.03 | 0.53 | 0.98 |

MMP: matrix metalloproteinase; NE, neutrophil elastase; NGAL, [neutrophil gelatinase-associated lipocalin](https://www.google.tn/url?sa=t&rct=j&q=&esrc=s&source=web&cd=1&cad=rja&uact=8&ved=0CCIQFjAA&url=http%3A%2F%2Fwww.ncbi.nlm.nih.gov%2Fpmc%2Farticles%2FPMC2528839%2F&ei=KaWZVdKjHoaxUYrlhfAH&usg=AFQjCNFIidM3Mj4RRIY0Wi31i5644Vt8hQ&bvm=bv.96952980,d.d24); IL-6, interleukin-6. (a) Comparisons of MMPs, proMMP-9/NGAL, NGAL, NE and IL-6 levels between healthy smokers and COPD smokers were performed using (a) the Mann–Whitney test, and (b) an analysis of covariance as a function of age and pack years.
