# Supplementary material for: Concomitant elevations of MMP‐9, NGAL, proMMP‐9/NGAL and neutrophil elastase in serum of smokers with chronic obstructive pulmonary disease
Source: J Cell Mol Med. 2016 Dec 22;21(7):1280–91. doi: 10.1111/jcmm.13057 (PMC5487915; doi:10.1111/jcmm.13057)
Supplement: Supplementary file 2 — Table S2 Mutual correlations among (pro and active) MMPs, proMMP‐9/NGAL, NGAL, NE and IL‐6 in serum from COPD smokers. [file JCMM-21-1280-s002.docx]

**Supplemental data 2**  Mutual correlations among (pro and active) MMPs, proMMP-9/NGAL, NGAL, NE and IL-6 in serum from

COPD smokers

|  | MMP-9 | NGAL | proMMP-9/NGAL | NE |
| --- | --- | --- | --- | --- |
| IL-6 | r=-0.231  p=0.054 | r=0.16  p=0.19 | r=-0.105  p=0.382 | r=0.175  p=0.24 |
| MMP-2 | r=-0.023  p=0.81 | r=-0.04  p=0.72 | r=-0.12  p=0.29 | r=-0.293  **p=0.04** |
| MMP-3 | r=-0.065  p=0.66 | r=0.044  p=0.76 | r=-0.01  p=0.95 | r=0.12  p=0.42 |
| MMP-12 | r=-0.05  p=0.78 | r=0.152  p=0.391 | r=-0.078  p=0.66 | r=0.07  p=0.703 |

Spearman correlation coefficient (r) and the p-value are shown. Bold p-values represent significant correlation.

MMP, matrix metalloproteinase; NE, neutrophil gelatinase; NGAL, neutrophil gelatinase-associated lipocalin;

IL-6, interleukin-6. ELISA assays determine pro and active MMPs concentrations in serum.
