# Supplementary material for: Concomitant elevations of MMP‐9, NGAL, proMMP‐9/NGAL and neutrophil elastase in serum of smokers with chronic obstructive pulmonary disease
Source: J Cell Mol Med. 2016 Dec 22;21(7):1280–91. doi: 10.1111/jcmm.13057 (PMC5487915; doi:10.1111/jcmm.13057)
Supplement: Supplementary file 3 — Table S3 Demographic and clinical characteristics of healthy and COPD smokers. [file JCMM-21-1280-s003.doc]

**Supplemental data 3** Demographic and clinical characteristics of healthy and COPD smokers

| **Parameter** | **Healthy**  **n=12** | **COPD**  **n=19** | **GOLD I/II**  **n=14** | **GOLD III/IV**  **n=5** |
| --- | --- | --- | --- | --- |
| **Age (years)**  **Male (%)**  **BMI**  **Pack years**  **FEV1**  **FEV1% pred**  **FVC**  **FVC % pred** | 57.65±1.34  100  27.63±0.88  18.05±4.05  3.06±0.18  93.26±1.93  3.71±0.21  95±2.18 | 60.31±1.4  100  27.4±1.46  47.12±4.57  1.76±0.16  58.21±4.69  3.07±0.18  80.37±3.95 | 59.85±1.63  100  27.47±1.67  49.77±2.64  2.04±0.15  67.78±3.52  3.25±0.19  86.21±3.51 | 61.6±2.97  100  27.19±3.49  39.7±7.03  0.98±0.13  31.4±4.31  2.56±0.33  64±8.02 |

Data are presented as mean ± SEM. COPD: chronic obstructive pulmonary disease; BMI: body mass index

[weight (kg) / height^2^ (m^2^)]; Pack years (number of cigarettes smoked per day x number of years smoked) /20;

FEV1: forced expiratory volume in 1 s, % pred: percent of predicted value; FVC: forced vital capacity.
