# Supplementary material for: Concomitant elevations of MMP‐9, NGAL, proMMP‐9/NGAL and neutrophil elastase in serum of smokers with chronic obstructive pulmonary disease
Source: J Cell Mol Med. 2016 Dec 22;21(7):1280–91. doi: 10.1111/jcmm.13057 (PMC5487915; doi:10.1111/jcmm.13057)

**Supplemental data 4**  RT-PCR analysis of MMP-2/-12 and IL-6 transcripts in PBMCs from healthy smokers and COPD smokers

Data are expressed as the ratio between the analyte transcript and the β2-microglobulin transcript. Values are reported as the median (IQR). (A) Values of MMP-2/-12 and IL-6 transcripts in healthy (n=12) and COPD (n=19) samples. (C) Values of MMP-2/-12 and IL-6 transcripts for GOLD stages (GOLD I/II, n=14; GOLD III/IV, n=5).


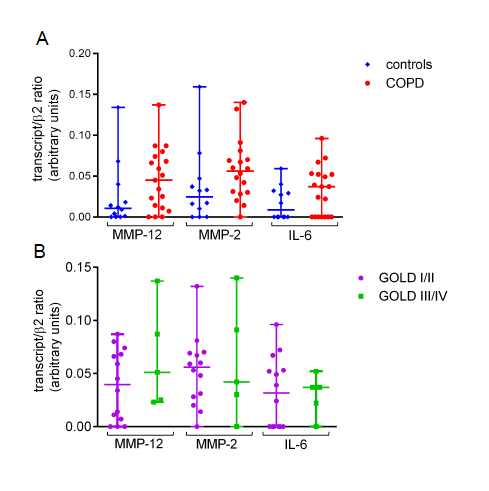

Supplement: Supplementary file 4 — Figure S1 RT‐PCR analysis of MMP‐2/‐12 and IL‐6 transcripts in PBMCs from healthy smokers and COPD smokers. [file JCMM-21-1280-s004.docx]
